# Ultrafast surface modification of Ni_3_S_2_ nanosheet arrays with Ni-Mn bimetallic hydroxides for high-performance supercapacitors

**DOI:** 10.1038/s41598-018-22448-w

**Published:** 2018-03-14

**Authors:** Xu Zou, Qing Sun, Yuxin Zhang, Guo-Dong Li, Yipu Liu, Yuanyuan Wu, Lan Yang, Xiaoxin Zou

**Affiliations:** 1 State Key Laboratory of Inorganic Synthesis and Preparative Chemistry, College of Chemistry, Jilin University, Changchun, 130012 P. R. China; 2 State Key Laboratory of Mechanical Transmissions, College of Material Science and Engineering, Chongqing University, Chongqing, 400044 P. R. China

## Abstract

Amorphous Ni-Mn bimetallic hydroxide film on the three-dimensional nickle foam (NF)-supported conductive Ni_3_S_2_ nanosheets (denoted as Ni-Mn-OH@Ni_3_S_2_/NF) is successfully synthesized by an ultrafast process (5 s). The fascinating structural characteristic endows Ni-Mn-OH@Ni_3_S_2_/NF electrodes better electrochemical performance. The specific capacitance of 2233.3 F g^−1^ at a current density of 15 A g^−1^ can achieve high current density charge and discharge at 20/30 A g^−1^ that the corresponding capacitance is 1529.16 and 1350 F g^−1^, respectively. As well as good cycling performance after 1000 cycles can maintain 72% at 15 A g^−1^. The excellent performance can be attributed to unique surface modification nanostructures and the synergistic effect of the bimetallic hydroxide film. The impressive results provide new opportunity to produce advanced electrode materials by simple and green route and this material is expected to apply in high energy density storage systems.

## Introduction

In recent years, with the increasing energy consumption and environmental degradation problems, great strive have been made to develop alternative energy sources and high-power energy storage systems^1,2^. Supercapacitors, known as a promising energy-storage device, have attracted widely interest due to their advantages of high-power capability, quick charge-discharge performance and long cycling lifetime^3–6^. Presently, most electrode materials for commercial supercapacitors are based on pure carbon (e.g. electric double-layer capacitive materials), which exhibits poor specific capacitance and cannot fulfill the ever-growing demands for high-energy storage applications. By comparison, pseudocapacitive materials based on transition metal oxides/hydroxides can obviously offer much higher specific capacitance and larger energy densities by using fast and reversible Faradaic reactions to store energy^7–10^. Particularly, nickel^11–14^, manganese-based^15–17^ hydroxides/oxides and their compounds^18–21^ have been recognized as promising electrode materials because of their low toxicity, low cost, great structures and morphology flexibility^22–28^. Unfortunately, they usually exhibit poor cycling stability and cannot tolerate charge/discharge at high current density due to their inferior conductivity which limits the electron transport.

To overcome this problem, building hierarchical architectures by growing the active materials on highly conductive backbone (such as carbon fiber, graphene) has been demonstrated to be a feasible strategy^11,29–32^. This is because the conductive backbone can severe as highly efficient electron highways and significantly enhance the rate capability of electrode materials. However, fabrication processes to obtain the hybrid hierarchical structures are usually tedious and costly, which limit their practical applications. Thus, developing a facile method for rapid synthesizing the freestanding hybrid nanostructures between pseudocapacitive materials and suitable conductive backbone is challenging but significant. Heazlewoodite Ni_3_S_2_, a kind of metal sulfide with a network of Ni-Ni metal bonds through the whole structure, has been proved to possess a well metallic conductivity^33–35^. What’s more, the surface of Ni_3_S_2_ can be easily modified by chemical treatment because of its abundant nucleation sites. Above features make Ni_3_S_2_ suitable for using as backbone materials^34^.

In this paper, we present a facile method of growing Ni-Mn bimetallic hydroxide (Ni-Mn-OH) films on Ni_3_S_2_ nanosheet arrays which supported by nickel foam (NF), achieving excellent electrochemical performance and cycling stability for supercapacitors. This reasonable design can achieve optimal electrochemical performance that the specific capacitance about 3588.8, 2233.3, 1529.16 and 1350 F g^−1^ at 10, 15, 20 and 30 A g^−1^, respectively. The cycle stability test of Ni-Mn-OH@Ni_3_S_2_/NF shows that the specific capacitance of the electrode maintains 72% at 15 A g^−1^ after 1000 cycles. To our best knowledge, these electrochemical performances of Ni-Mn-OH@Ni_3_S_2_/NF are very outstanding in the reported supercapacitor systems and these properties suggested a logical experimental method for improving the supercapacitors performance.

## Results and Discussion

**Figure 1:**
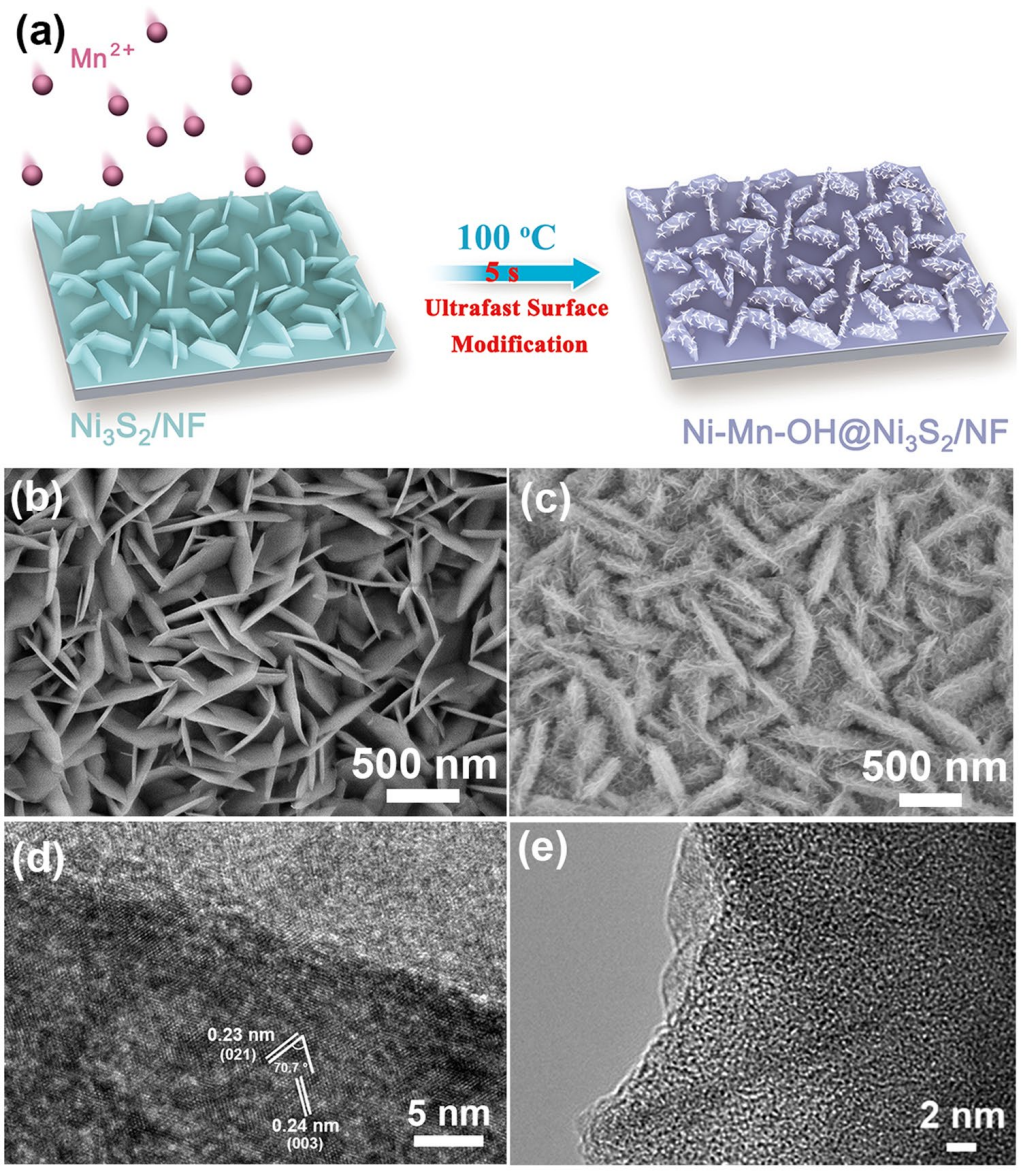
(**a**) Schematic illustration of the synthesis of Ni-Mn-OH@Ni_3_S_2_/NF by rapid immersing Ni_3_S_2_/NF in a 100 °C pre-heated aqueous solution with MnCl_2_ for 5 s. SEM images of (**b**,**c**) Ni_3_S_2_/NF and Ni-Mn-OH@Ni_3_S_2_/NF, respectively. (**d**,**e**) HRTEM images of Ni-Mn-OH@Ni_3_S_2_/NF.

To synthesize the Ni-Mn-OH@Ni_3_S_2_/NF, an ultrafast (5 s) surface modification method was conducted (see Fig. 1a and Experimental section for details). The whole synthesis process is rapid and easy to operate without using any template that is supposed to suitable for large-scale synthesis. We can simply think that this so-called ultrafast surface modification reaction is based on the classical solid-liquid interface nucleation growth theory^36^. The scanning electron microscopy (SEM) images of Ni_3_S_2_ nanosheet arrays on the NF are shown in Figs 1b and S1. It is obversed that the entire surface of NF is completely wrapped by the Ni_3_S_2_ nanosheets. Ni_3_S_2_ nanosheets has smooth surface and vertical growth on NF. The thickness is around 20–30 nm. Apparently, after the rapid reaction of interface nucleation, the Ni-Mn-OH film can be observed to grow on the Ni_3_S_2_ nanosheets (Fig. 1c). The Ni-Mn-OH film is composed of many nanoflakes and the thickness is approximately 10–12 nm. We can observed that the Ni-Mn-OH film completely covered on the surface of Ni_3_S_2_/NF, finally leading to the formation of a hierarchical Ni-Mn-OH@Ni_3_S_2_/NF nanostructure. Futher structural details of Ni-Mn-OH@Ni_3_S_2_/NF were displayed in high resolution transmission electron microscopy (HRTEM). The HRTEM images of Ni-Mn-OH@Ni_3_S_2_/NF show a completely different crystallinity from Fig. 1d. In the better crystallinity area, we can observe that two kinds of lattice spacings are 0.23 nm and 0.24 nm. The two lattice spacings corresponding interplanar angle is about 70.7°. These features are consistent with the (021) and (003) crystallographic planes of hexagonal Ni_3_S_2_ phase. In contrast, an amorphous film can be obviously observed on the other side. These results indicate that the Ni-Mn-OH film grow on Ni_3_S_2_ is in an amorphous state. The amorphous phase is also supported by transmission electron microscopy (TEM) in Fig. 1e.

**Figure 2:**
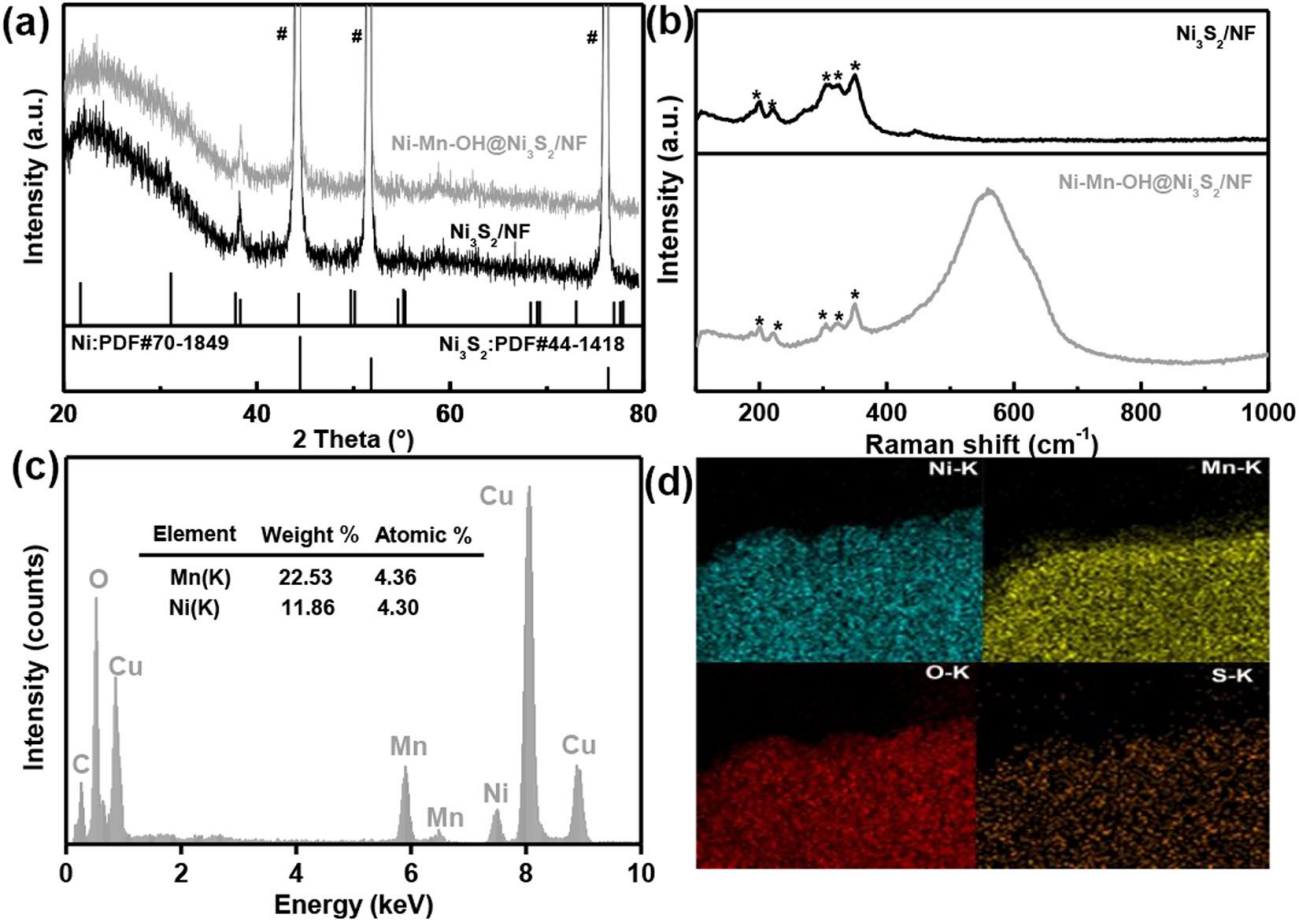
(**a**) XRD patterns and (**b**) Raman spectra of Ni-Mn-OH@Ni_3_S_2_/NF and Ni_3_S_2_/NF. (**c**) STEM-EDS spectrum of the amorphous Ni-Mn-OH region. The Cu peaks in the EDS spectrum come from the Cu grid as sample holder. (**d**) The corresponding elemental mapping images.

Figure 2a displays the X-ray diffraction (XRD) patterns of Ni-Mn-OH@Ni_3_S_2_/NF and Ni_3_S_2_/NF. Apparently, these two materials have the similar XRD patterns, which belong to the hexagonal Ni_3_S_2_ (JCPDS card No.44-1418) and metallic nickel (JCPDS card No.70-1849) without additional peaks present. The result further reveals that Ni-Mn-OH is an amorphous film. The Raman spectra was performed over the rang of 100–1000 cm^−1^ to demonstrate the film composition (Fig. 2b). Ni-Mn-OH@Ni_3_S_2_/NF and Ni_3_S_2_/NF have the same Raman bands with Ni_3_S_2_ at 188, 198, 221, 303, 322 and 349 cm^−1^ (labelled by “*”)^37^. However, Ni-Mn-OH@Ni_3_S_2_/NF appears additional broad Raman bands, which is belong to the amorphous Ni-Mn-OH film. There are two obvious bands are observed at 560 cm^−1^ and 630 cm^−1^. The Raman band at 560 cm^−1^ can be attributed to the Ni-O vibrations^38^. Obviously, Ni-O vibration values of Ni-Mn-OH@Ni_3_S_2_/NF are a little more wider than those of pure Ni(OH)_2_ clusters which is related to the disordered Ni(OH)_2_^39,40^. The typical Raman peak at around 630 cm^−1^ is corresponding to the Mn-O vibrations in MnOOH clusters^41^. The above results show that the film is composed of Ni-Mn-OH. To gain further information of the composition, we empolyed X-ray photoelectron spectroscopy (XPS) spectra to detect the elements valence state in the Ni-Mn-OH film (Fig. S2). From the Ni 2p XPS spectrum shown in Fig. S2a, we can observe two major peaks with binding energies at 856.2 eV and 874.1 eV correspond to Ni 2p3/2 and Ni 2p1/2 spin-orbit peaks, respectively^42,43^. The result shows that the oxidation state of Ni in the film is dominated by 2+. In addition, the Mn 2p1/2 and Mn 2p3/2 spin-orbit peaks are located at 654.5 and 642.7 eV, suggesting the presence of Mn^3+^ oxidation state in the sample (Fig. S2b)^44–46^. No signal of S is detected, further confirming that there is absence of S species in the amorphous Ni-Mn-OH film. The scanning transmission electron microscopy energy-dispersive X-ray spectroscopy (STEM-EDS) is used to characterize the composition of the Ni-Mn-OH film (Fig. S3). The STEM-EDX spectrum result indicates that the atomic ratio of Ni and Mn is 1:1.25 in Ni-Mn-OH film and no S exist (Fig. 2c). Futhermore, the corresponding element mapping analysis of Ni-Mn-OH@Ni_3_S_2_/NF is shown in the Fig. 2d. The Ni, Mn, O uniformly distributed throughout the whole hybrid and S is mainly distributed on the Ni_3_S_2_/NF. The result illustrated that the film is composed of Ni and Mn. The nickel in the Ni-Mn-OH film mainly comes from the inside of the Ni_3_S_2_/NF. In summary, the inner and outer nanoframes are derived from the Ni_3_S_2_ and the Ni-Mn-OH film, respectively.

**Figure 3:**
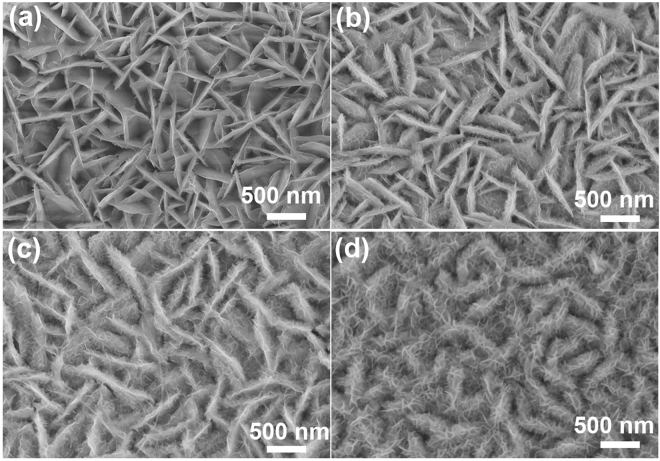
SEM images of Ni-Mn-OH@Ni_3_S_2_/NF with different concentration of manganese (**a**) NiMn_0.249_OH@Ni_3_S_2_/NF, (**b**) NiMn_0.279_OH@Ni_3_S_2_/NF, (**c**) NiMn_0.968_OH@Ni_3_S_2_/NF and (**d**) Ni_2.73_MnOH@Ni_3_S_2_/NF.

In order to better understand the film formation process, we investigated the impact of concentration of Mn on the morphology (Fig. 3a–d). When Mn precursor was used, Ni-Mn bimetallic hydroxide nanostructure is formed. The amorphous film morphology can’t form until the amount of MnCl_2_ was increased up to 0.369 mmol. When the amount of MnCl_2_ was further increased to 1.85 mmol and 5.53 mmol, the thickness of the nanoflakes was changed from 9 to 18 nm. The atomic ratio of Ni: Mn in the amorphous Ni-Mn bimetallic hydroxide film increased from 1: 0.249 to 1: 2.73 by changing the mass of MnCl_2_. When we directly immerse NF in a 100 °C pre-heated aqueous solution with manganese ions and sodium nitrate, there is no substance on the NF surface (Fig. S4a,b). This result suggested that the nickel in the film is mainly derived from Ni_3_S_2_ nanosheets. With the further increase in concentration of manganese ions, the morphology of Ni-Mn-OH film becomes bigger and thicker. When the amount of MnCl_2_ increased to 7.46 mmol, we found that the Ni_3_S_2_ nanosheets disappeared (Fig. S5). We also studied the effect of reaction time on the structure and electrochemical properties. When the reaction time is 2 s, only a small amount of Ni-Mn-OH is formed (Fig. S6a). As the reaction time prolongs, the sheet gradually becomes larger and wraps Ni_3_S_2_. When the time is up to 1 min, we cannot see the Ni_3_S_2_ (Fig. S6b–d). The above results overall indicate that the amorphous Ni-Mn bimetallic hydroxide films is *in situ* formed on the surface of Ni_3_S_2_ nanosheets after the unltrafast surface modification. Moreover, it should be mentioned that the presence of Mn precursor play vital roles in the morphology of film and composition for the reaction product, also affects supercapacitor performances.

**Figure 4:**
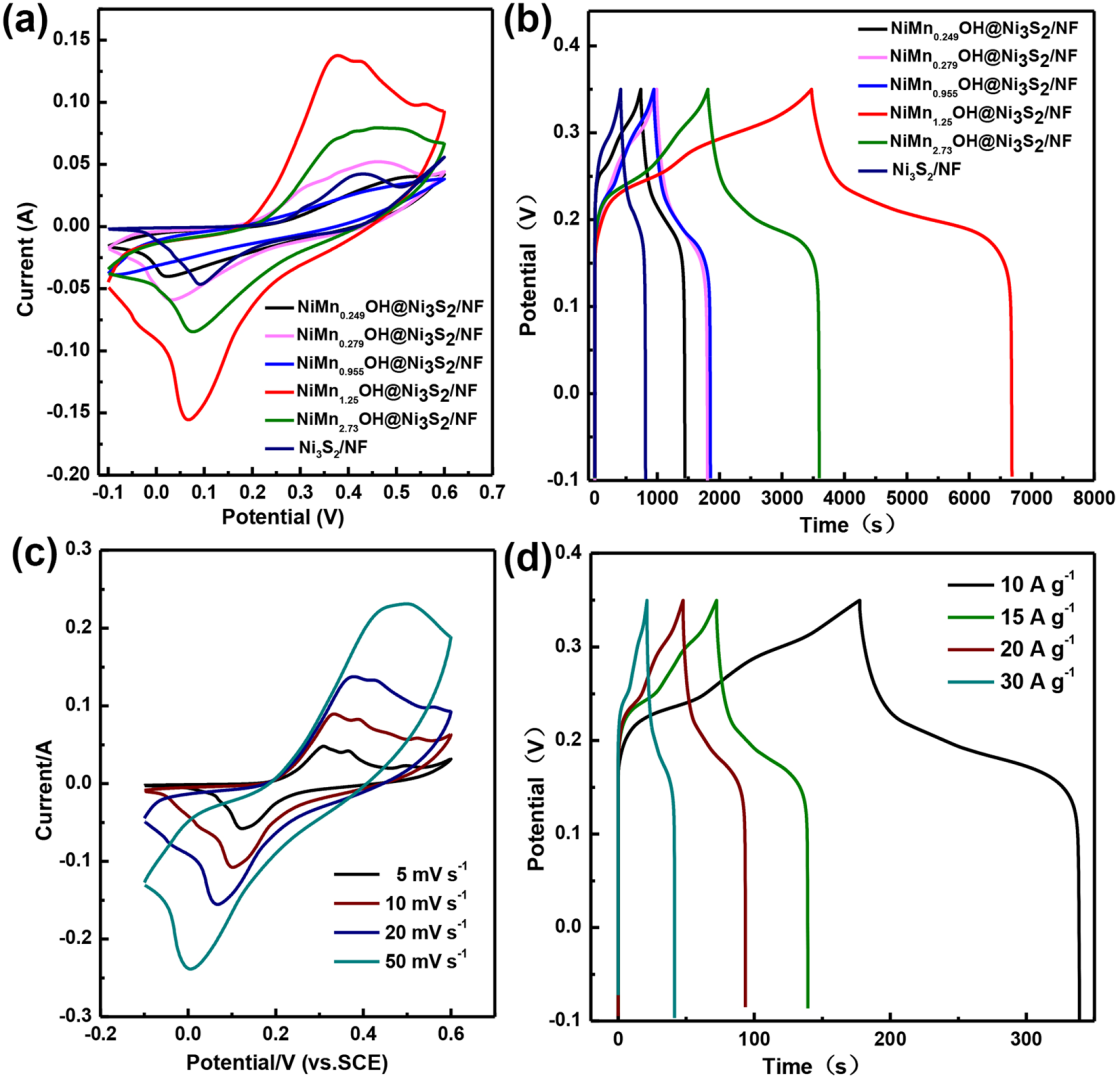
The contrast tests of six electrodes: (**a**) Cyclic voltammograms at a scan rate of 20 mV s^-1^ in 1 M KOH aqueous electrolyte. (**b**) Galvanostatic charge-discharge at a current density 1 A g^−1^. Electrochemical performances of NiMn_1.25_OH@Ni_3_S_2_/NF: (**c**) Cyclic voltammograms at different scan rates in 1 M KOH aqueous electrolyte. (**d**) Galvanostatic charge-discharge at different current density (the inset shows the specific capacitance under different current densities).

The electrochemical properties of Ni-Mn-OH@Ni_3_S_2_/NF which have the novel complex structure were investigated in 1 M KOH solutions. This is implementing by using Ni-Mn-OH@Ni_3_S_2_/NF directly as working electrode in a typical three-electrode electrochemical system (see details in the Experimental Section). First, electrochemical tests were performed on different reaction times. The area of cyclic voltammetry (CV) curves of the 5 s is the largest, indicating the better electrochemical propertie (Fig. S7). Then we compared several reference materials including Ni_3_S_2_/NF, NiMn_0.249_OH@Ni_3_S_2_/NF, NiMn_0.279_OH@Ni_3_S_2_/NF, NiMn_0.955_OH@Ni_3_S_2_/NF, NiMn_1.25_OH@Ni_3_S_2_/NF and NiMn_2.73_OH@Ni_3_S_2_/NF at the same conditions (Fig. 4a,b). Figure 4a shows representative CV curves of the electrodes in a voltage window of −0.1 to 0.6 V versus a standard calomel electrode (SCE). The shape of obtained CV curves is different from that of electric double-layer capacitance, showing obvious pseudocapacitive characteristics and exhibit highly reversible redox peaks. The highest currents and enclosed area of the NiMn_1.25_OH@Ni_3_S_2_/NF are much higher than other materials. Figure 4b shows the galvanostatic charge/discharge measurements performed in a voltage range between −0.1 and 0.6 V (vs SCE) at current densities 10 A g^−1^. NiMn_1.25_OH@Ni_3_S_2_/NF possesses longer discharge time, which is consistent with the results of the CV measurements. The specific capacitances of Ni_3_S_2_/NF NiMn_0.249_OH@Ni_3_S_2_/NF, NiMn_0.279_OH@Ni_3_S_2_/NF, NiMn_0.955_OH@Ni_3_S_2_/NF, NiMn_1.25_OH@Ni_3_S_2_/NF and NiMn_2.73_OH@Ni_3_S_2_/NF are about 886.7 F g^−1^, 1570 F g^−1^, 1853.3 F g^−1^, 2016.4 F g^−1^, 7132 F g^−1^ and 3968 F g^−1^ at 1 A g^−1^, respectively. The results mean that the capacitance of NiMn_1.25_OH@Ni_3_S_2_/NF has increased eight times compared with that of Ni_3_S_2_/NF. Moreover, the CV curve area of NiMn_1.25_OH@Ni_3_S_2_/NF is larger than that of the others at the identical scan rate of 20 mV s^−1^, and discharge times of NiMn_1.25_OH@Ni_3_S_2_/NF electrode is the longest compared to the other five electrodes. These results overall confirm that NiMn_1.25_OH@Ni_3_S_2_/NF electrode possesses a significantly enhanced specific capacitance compared with Ni_3_S_2_/NF electrode, indicating the important role of amorphous Ni-Mn-OH layer in electrochemical properties.

To further demonstrate that NiMn_1.25_OH@Ni_3_S_2_/NF has the best electrochemical performance, the Electrochemical Impedance Spectra (EIS) tests were conducted. It is a supercapacitor performance indicator that determines the dielectric properties of materials. Fig. S8 shows the electron conductivity comparison of NiMn_0.249_OH@Ni_3_S_2_/NF, NiMn_0.279_OH@Ni_3_S_2_/NF, NiMn_0.955_OH@Ni_3_S_2_/NF, NiMn_1.25_OH@Ni_3_S_2_/NF and NiMn_2.73_OH@Ni_3_S_2_/NF electrodes. In the curve, the internal resistance (R_s_) is the intersection with X-axis at high frequency, and the charge transfer resistance (R_CT_) is the diameter of the semicircle. The internal resistances (R_s_) for NiMn_0.249_OH@Ni_3_S_2_/NF, NiMn_0.279_OH@Ni_3_S_2_/NF, NiMn_0.955_OH@Ni_3_S_2_/NF, NiMn_1.25_OH@Ni_3_S_2_/NF and NiMn_2.73_OH@Ni_3_S_2_/NF electrodes are about 3.2, 1.3, 4.6, 0.6 and 1.2 Ω, respectively. We can see that total internal resistance of NiMn_1.25_OH@Ni_3_S_2_/NF electrode system is the smallest (0.6 Ω) and the R_ct_ of NiMn_1.25_OH@Ni_3_S_2_/NF is also the smallest. These demonstrate the dielectric properties of the material are favorable. Besides, the NiMn_1.25_OH@Ni_3_S_2_/NF electrode exhibits an almost vertical line along the imaginary axis in the low-frequency region, indicating its ideally capacitive behavior. The lowest R_ct_ of NiMn_1.25_OH@Ni_3_S_2_/NF demonstrates a facile charge transfer process at the electrode/electrolyte interfaces, which is beneficial to obtain a better electrochemical performance.

In order to better investigate the electrochemical properties of the NiMn_1.25_OH@Ni_3_S_2_/NF electrode, the three-electrode systematic tests are carried out. The redox peak can be observed in each CV curves, indicating that the measured capacitance is mainly based on the pseudocapacitive nature (Fig. 4c)^47,48^. The CV curves of the NiMn_1.25_OH@Ni_3_S_2_/NF electrode are not ideal rectangular. Because of the reversible Faradaic reaction of Ni^3+^ in 1 M KOH solution, a pair of redox peaks over the entire range is clearly observed, suggesting an ideal capacitive behavior of Ni-Mn-OH film. From the Fig. 4, about the redox reactions of the composite electrode in 1 M KOH electrolyte, the oxidizing reaction is at 0.4 V, and the reduction reaction is at 0.1 V, which can be represented by the following electrochemical reaction^49^:

$${\rm{Ni}}{({\rm{OH}})}_{{\rm{2}}}+{{\rm{OH}}}^{-}={\rm{NiOOH}}+{{\rm{H}}}_{{\rm{2}}}{\rm{O}}+{{\rm{e}}}^{-}$$

And for the multicomponent Ni-Mn-OH oxyhydroxide, Mn doping can extend the effective potential window^50^. The shape of the CV curve has not altered much with the increase of scan rate, revealing remarkable mass transport of electrons and ions. Furthermore, the nearly symmetrical triangle shapes of the charge/discharge curves of the electrode at various current densities were also collected to further evaluate the electrochemical performance in Fig. 4d. By calculation, the specific capacitances of the electrode are about 3588.8 and 2233.3 F g^−1^ at 10 and 15 A g^−1^, respectively. More importantly, we can also see NiMn_1.25_OH@Ni_3_S_2_/NF electrode is suitable for high current charge/discharge. The specific capacitance of the electrode remains at 1529.16 and 1350 F g^−1^ at a high current density of 20 and 30 A g^−1^, respectively.

**Figure 5:**
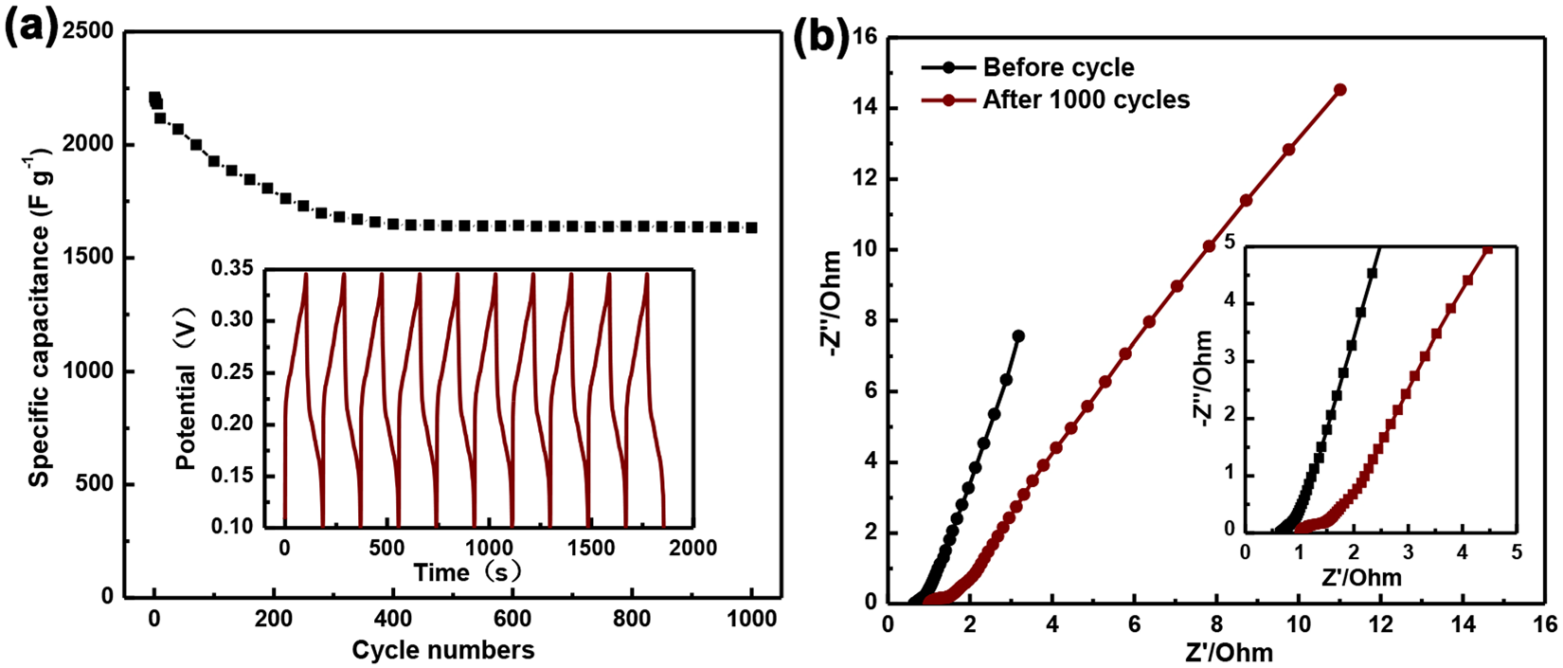
(**a**) Cycling performance at the current density of 15 A g^−1^ (the inset shows the galvanotactic charge–discharge curves of the last 10 cycles). (**b**) The comparison of Nyquist plot: initial Nyquist plot and Nyquist plot after 1000 cycling.

Cyclic stability is an important factor that should be considered when it refers to the application of supercapacitor materials in the energy field. To assess the cycling performance of the NiMn_1.25_OH@Ni_3_S_2_/NF at a current density of 15 A g^−1^ is shown in Fig. 5a. Impressively, the NiMn_1.25_OH@Ni_3_S_2_/NF show outstanding capacitance retention of maintains 72% after 1000 cycles. Additionally, we can be seen from the electrochemical impedance spectra of the NiMn_1.25_OH@Ni_3_S_2_/NF electrode before and after 1000 cycles (Fig. 5b), the internal resistance (R_s_) of the NiMn_1.25_OH@Ni_3_S_2_/NF electrode changes from 0.6 Ω to 1.0 Ω, which has only a slightly increase after such long time cyclic stability tests. This result implies that there is a peaceable redox reaction between the electrode and the electrolyte. During cycling, Ni^3+^ would be reduced to Ni^2+^ in alkaline solution and led to the destruction of structure, the attenuation of capacitance and finally maintained modest capacity.

In this work, the specific capacitance of NiMn_1.25_OH@Ni_3_S_2_/NF electrode exhibits more than eight times improvement at 1 A g^−1^ compared to the Ni_3_S_2_/NF electrode The enhanced specific capacitance of the NiMn_1.25_OH@Ni_3_S_2_/NF toward supercapacitors is interpreted as follows: (i) The Ni_3_S_2_ material becomes rougher by modificated of new capacitive material and further enlarge the specific surface area. Highly active surface area could improve the performance of supercapacitors. (ii) The bimetallic hydroxide with both nickel ion and manganese ion has various valence states to provide richer redox reactions compared with single-component hydroxide, resulting higher specific capacitance. (iii) Ni_3_S_2_ can support the outer structure of Ni-Mn-OH film to improve the stability during electrochemical cycling. Moreover, building binder free nanostructure would avoid reducing the electrical conductivity. (iv) The synergistic effect of conductive metal sulfide (Ni_3_S_2_/NF) and amorphous Ni-Mn bimetallic hydroxide film. The Ni_3_S_2_ and NiMn hydroxides interfaces form some heterojunctions, which can affect the charge transport/separation and storage at interface^51–53^. Ni_3_S_2_/NF, as backbones for supporting Ni-Mn-OH nanostructure, provide a highly efficient electron highways to enhance the capacitive performance.

## Conclusions

In summary, we have successfully prepared amorphous Ni-Mn-OH grown on Ni_3_S_2_ nanosheets (Ni-Mn-OH@Ni_3_S_2_/NF) by an ultrafast and efficient surface modification route. In view of the unique architecture and intrinsic properties, Ni-Mn-OH@Ni_3_S_2_/NF shows excellent electrochemical performance with specific capacitance of 3588.8 and 2233.3 F g^−1^ at a current density of 10 and 15 A g^−1^, respectively and good cycling performance (75% retention after 1000 cycles at 15 A g^−1^). Contrast with Ni_3_S_2_/NF, its performance improved eight times. The main reason is the synergistic effect between Ni_3_S_2_ nanosheets and amorphous Ni-Mn-OH film. We believe that the facile and novel synthesis strategy could be widespread used to fabrication of other binary or ternary metal oxides or hydroxides and Ni-Mn-OH@Ni_3_S_2_/NF as an ideal candidate material will be applied to supercapacitors and other energy-storage devices.

## Methods

### Synthesis of Ni_3_S_2_/NF

A piece of Ni foam (1 × 3 cm) was cleaned ultrasonically in acetone (15 mL) and then 3 M HCl solution (15 mL) for 10 min each, and washed subsequently with water and ethanol for several times. The cleaned Ni foam was submerged into a 25 mL Teflon-lined stainless autoclave containing 10 mL of 1.445 mmol/L thiourea solution. The autoclave was sealed and maintained at 150 °C for 5 h. The resulting material was washed with ethanol for three times and dried in vacuum at room temperature, leading to Ni_3_S_2_/NF.

### Synthesis of Ni-Mn-OH@Ni_3_S_2_/NF

0.739 g (3.73 mmol) Manganese(II) chloride hexahydrate (MnCl_2_·4H_2_O) and 0.212 g (2.49 mmol) sodium nitrate was dissolved in 50 mL of water at 100 °C for 5 min. Then Ni_3_S_2_/NF was immersed into the solution maintained at 100 °C for 5 s. The resulting material was washed with distilled water and ethanol for several times then dried at ambient temperature. For comparison, when the concentration of Mn^2+^ ions in the pre-heated solution was tuned, a series of Ni-Mn-OH@Ni_3_S_2_/NF with different Ni:Mn atomic ratios were synthesized (NiMn_0.249_OH@Ni_3_S_2_/NF, NiMn_0.279_OH@Ni_3_S_2_/NF, NiMn_0.955_OH@Ni_3_S_2_/NF, NiMn_1.25_OH@Ni_3_S_2_/NF and NiMn_2.73_OH@Ni_3_S_2_/NF).

### Structural characterization

The powder X-ray diffraction (XRD) patterns were recorded on a Rigaku D/Max 2550× -ray diffractometer with Cu Kα radiation (λ = 1.5418 Å). The X-ray photoelectron spectroscopy (XPS) was performed on an ESCALAB 250× -ray photoelectron spectrometer with a monochromatic X-ray source (Al Kα hυ = 1486.6 eV). The Raman spectra were obtained with a Renishaw Raman system model 1000 spectrometer with a 20 mW air-cooled argon ion laser (514.5 nm) as the exciting source. The transmission electron microscope (TEM) images were obtained with a Philips-FEI Tecnai G2S-Twin microscope equipped with a field emission gun operating at 200 kV. The scanning electron microscope (SEM) images were obtained with a JEOL JSM 6700 F electron microscope. Inductively coupled plasma atomic emission spectroscopy (ICP-OES) was performed on a Perkin-Elmer Optima 3300DV ICP spectrometer.

### Electrochemical measurements

All the electrochemical performance in three-electrode configurations was carried out on the CHI 660E electrochemical station. The Electrochemical properties were investigated in an aqueous KOH electrolyte (1 M) at room temperature. The three-electrode system consists of two loops, one circuit consists of a working electrode and a reference electrode, which is used to test the electrochemical reaction process of the working electrode. The other circuit is composed of a working electrode and an auxiliary electrode. The samples that size is 1 × 1 cm were directly used as the working electrode, a Pt plate as the counter electrode, and a standard calomel electrode (SCE) as the reference electrode. CV measurements were performed in the voltage window between −0.1 and 0.6 V at different scan rates. Galvanostatic charge–discharge experiments were performed by the potential from −0.1 V to 0.6 V at different current densities. The specific capacitances (C_m_) are calculated according to the following equation:

$${C}_{m}=\frac{{\rm{I}}{\rm{\Delta }}{\rm{t}}}{{\rm{m}}{\rm{\Delta }}{\rm{V}}}$$

Where I is the discharge current (A), ${\rm{m}}$ is the weight (g) of active materials, Δt is the discharge time (s), and ΔV is the discharging potential window (V)^54,55^. And the mass of active materials of NiMn_0.249_OH@Ni_3_S_2_/NF, NiMn_0.279_OH@Ni_3_S_2_/NF, NiMn_0.955_OH@Ni_3_S_2_/NF, NiMn_1.25_OH@Ni_3_S_2_/NF and NiMn_2.73_OH@Ni_3_S_2_/NF are calculated as 0.8509 mg cm^−2^, 0.8577 mg cm^−2^, 0.906 mg cm^−2^, 0.9269 mg cm^−2^, 0.9699 mg cm^−2^, respectively. Electrochemical impedance spectroscopy (EIS) measured by the external field’s interaction with the dipole moment of a particular sample, usually stated by permittivity. EIS measurements were carried out by applying an alternating current (AC) voltage with 1 mV amplitude in a frequency range from 0.1 Hz to 100 KHz at open circuit potential. The cyclic stability was evaluated by cyclic voltammetry measurement at a current density of 15 A g^−1^ for 1000 cycles.

## Electronic supplementary material

Supplementary Information
