# Supplementary material for: Ultrafast surface modification of Ni3S2 nanosheet arrays with Ni-Mn bimetallic hydroxides for high-performance supercapacitors
Source: Sci Rep. 2018 Mar 14;8:4478. doi: 10.1038/s41598-018-22448-w (PMC5852135; doi:10.1038/s41598-018-22448-w)
Supplement: Supplementary file 1 — Supplementary Information [file 41598_2018_22448_MOESM1_ESM.doc]

**Supplementary Information**

**Ultrafast** **surface modification of Ni_3_S_2_ nanosheet arrays with Ni-Mn bimetallic hydroxides for high-performance supercapacitors**

Xu Zou^1+^, Qing Sun^2+^, Yuxin Zhang^2^,*, Guo-Dong Li^1^, Yipu Liu^1^, Yuanyuan Wu^1^, Lan Yang^1^ & Xiaoxin Zou^1^,*

^1^ State Key Laboratory of Inorganic Synthesis and Preparative Chemistry, College of Chemistry, Jilin University, Changchun 130012, P. R. China

^2^ State Key Laboratory of Mechanical Transmissions, College of Material Science and Engineering, Chongqing University, Chongqing 400044, P. R. China

*E-mail address: [xxzou@jlu.edu.cn](mailto:xxzou@jlu.edu.cn) (X. Z.), [zhangyuxin@cqu.edu.cn](mailto:zhangyuxin@cqu.edu.cn) (Y. Z.)

***
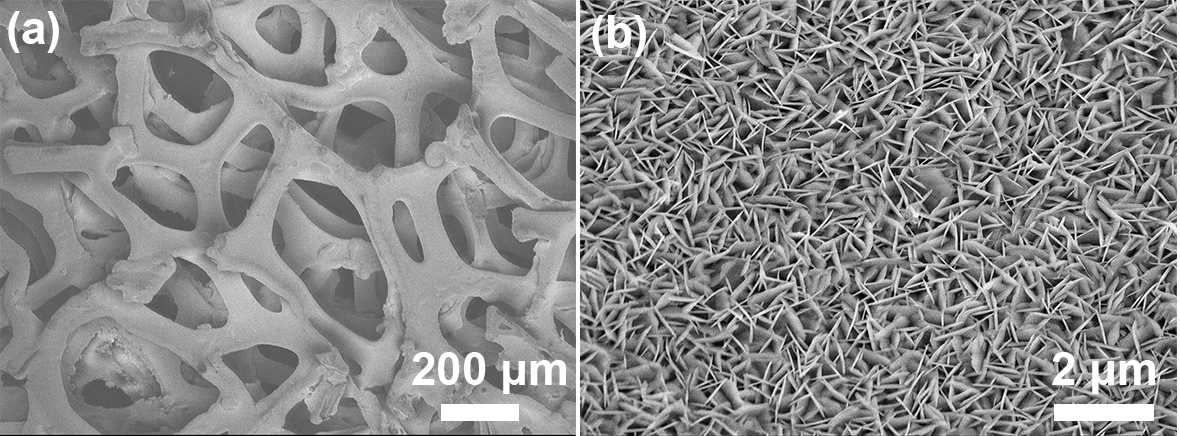
***


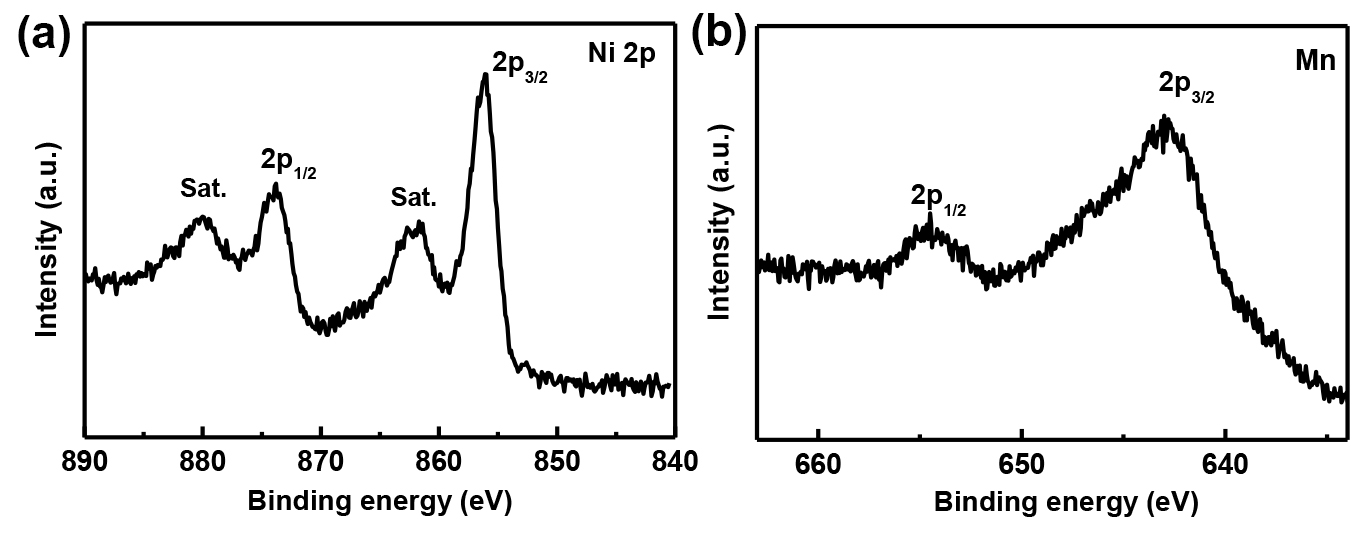
**Figure S1.** SEM images of Ni_3_S_2_/NF.


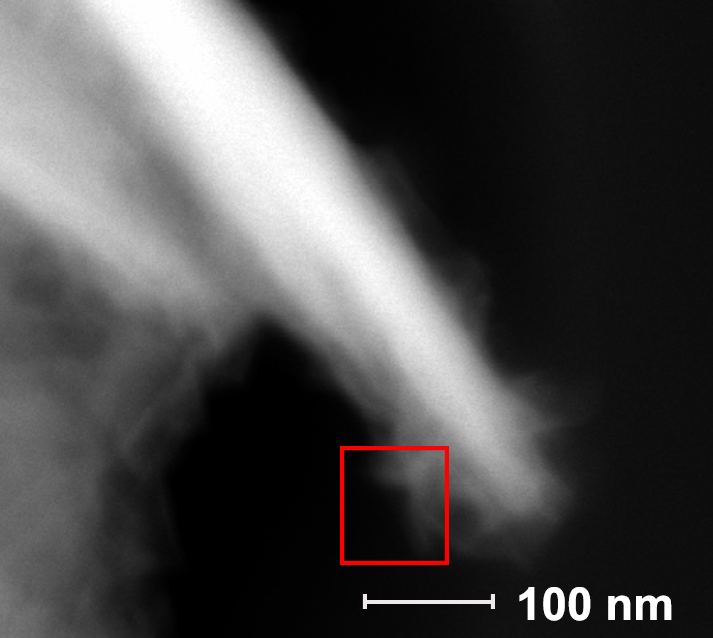
**Figure S2.** X-ray photoelectron spectra of Ni-Mn-OH@Ni_3_S_2_/NF. (a) Ni 2p, (b) Mn 2p.

**Figure S3.** STEM-EDX image of NiMn_1.25_OH @Ni3S2/NF.

**
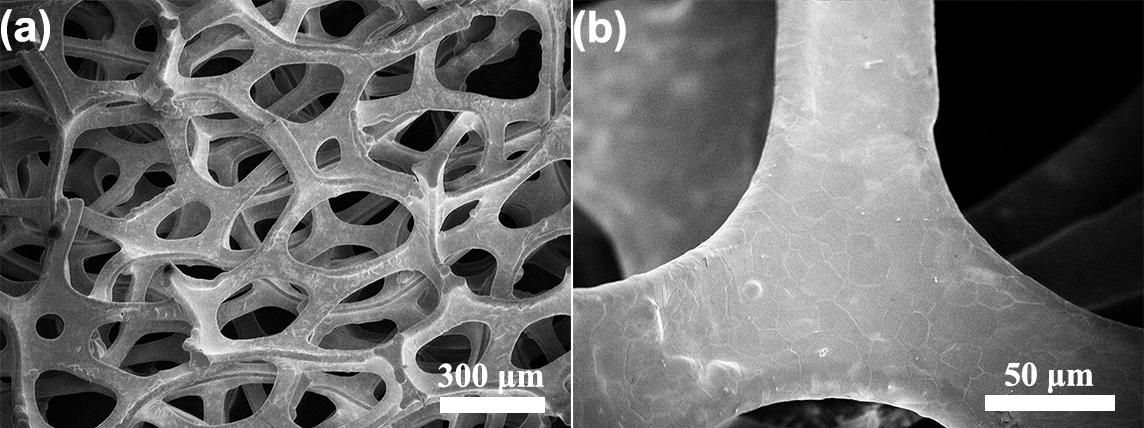
**


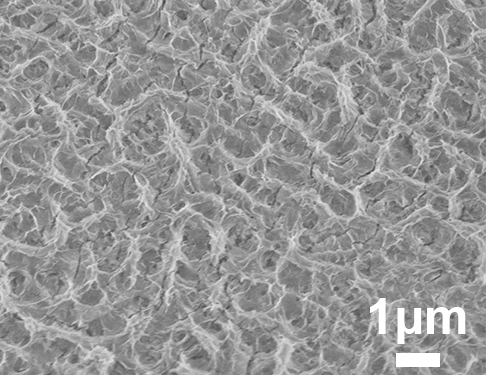
**Figure S4.** SEM images of NF in a 100 ^o^C pre-heated aqueous solution with manganese ions and sodium nitrate.


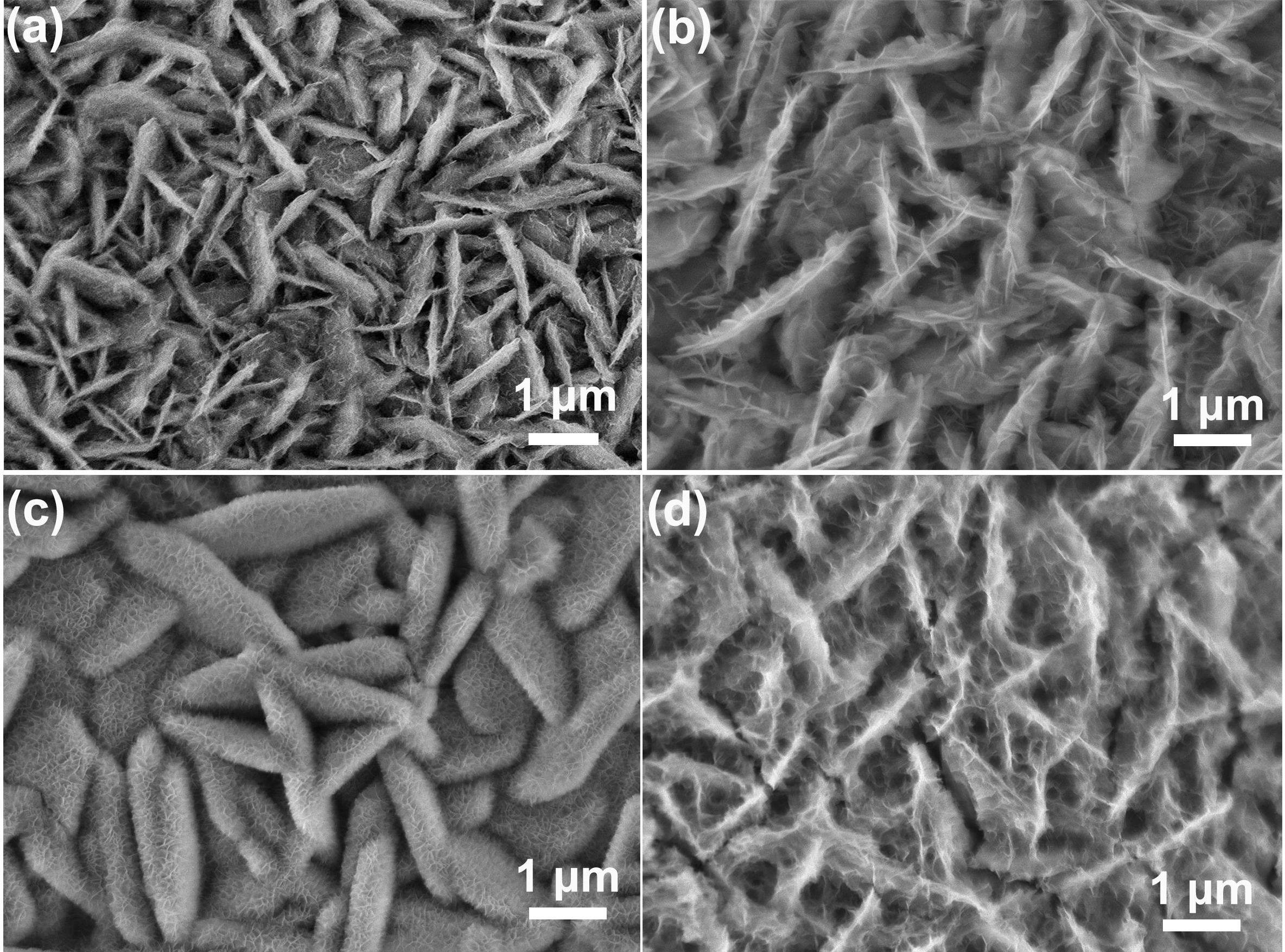
**Figure S5.** SEM images of the amount of 7.46 mmol of MnCl_2_

**Figure S6.** SEM images of Ni-Mn-OH@Ni_3_S_2_/NF with different reaction durations (a) 2 s, (b) 10 s, (c) 30 s and (d) 1 min


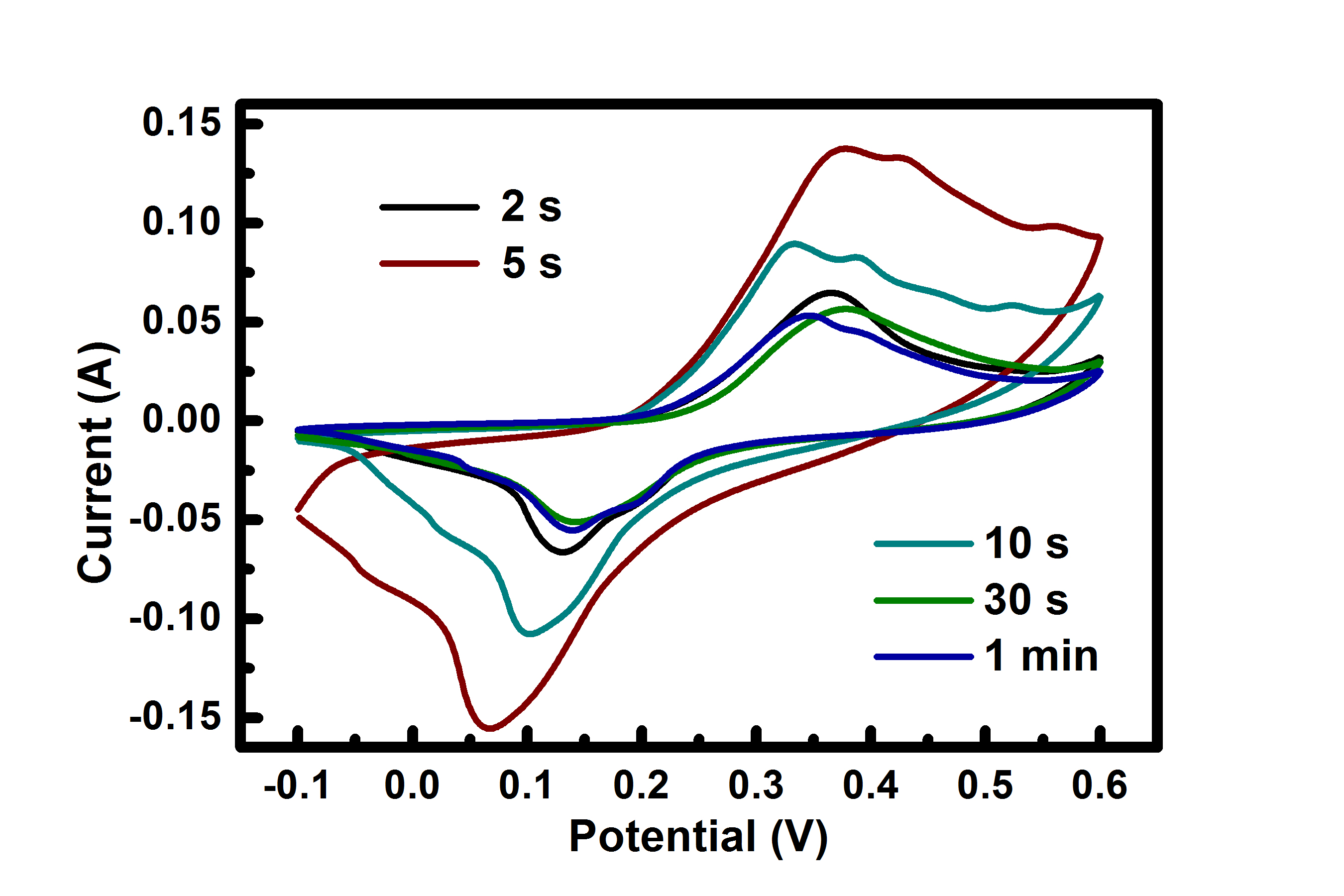

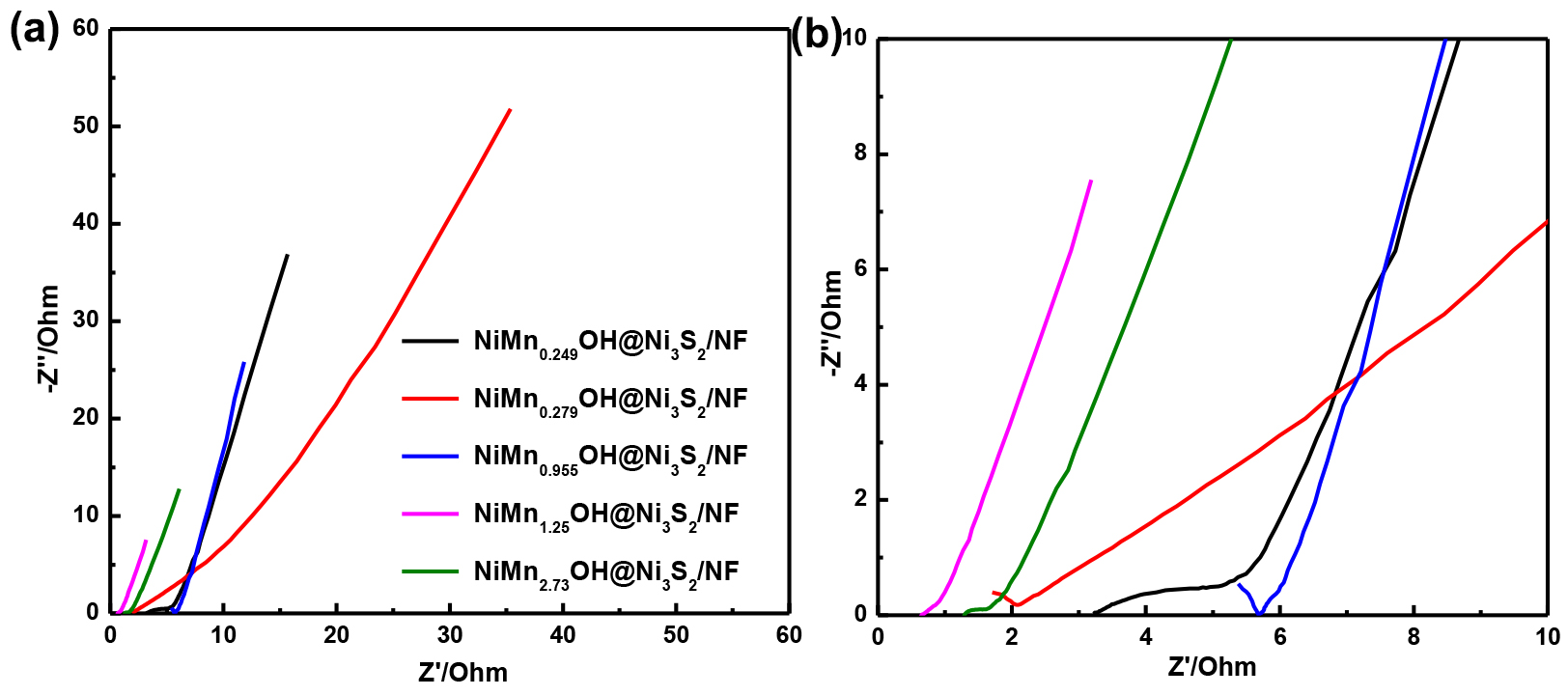
**Figure S7.** The CV curves at 10 mV s^-1^ of Ni-Mn-OH@Ni_3_S_2_/NF with different reaction durations: 2 s, 5 s,10 s, 30 s and 1 min.

**Figure S8.** (a) The comparison of Nyquist plot about different content of Mn. (b) Drawing of Partial Enlargement.

**Table S1.** Comparison of Ni:Mn atomic ratios with different manganese contents.

| C_(Mn_^2+^)/(mmol /L) | 1.47 | 7.47 | 37.28 | 74.68 | 111.97 |
| --- | --- | --- | --- | --- | --- |
| Ni:Mn | 1:0.249 | 1:0.279 | 1:0.955 | 1:1.25 | 1:2.73 |
